# Epigenetic Regulation of Salt Stress Responses in Rice: Mechanisms and Prospects for Enhancing Tolerance

**DOI:** 10.3390/epigenomes9040046

**Published:** 2025-11-16

**Authors:** Emanuela Talarico, Eleonora Greco, Francesco Guarasci, Fabrizio Araniti, Adriana Chiappetta, Leonardo Bruno

**Affiliations:** 1 Department of Biology, Ecology and Earth Sciences (DiBEST), Unit of Plant Biology, University of Calabria, 87036 Rende, Italy; emanuela.talarico@unical.it (E.T.); eleonora.greco@unical.it (E.G.); francesco.guarasci@unical.it (F.G.); adriana.chiappetta@unical.it (A.C.); 2 Department of Agricultural and Environmental Sciences—Production, Landscape, Agroenergy (Di.S.A.A.), University of Milano, 20133 Milano, Italy; fabrizio.araniti@unimi.it

**Keywords:** salt stress, *Oryza sativa*, epigenetic regulation, DNA methylation, histone modification, non-coding RNA, chromatin remodelling, salinity tolerance, breeding strategies, wild rice species

## Abstract

Rice (*Oryza sativa* L.) is a staple food for over half the global population and a model organism for monocot plant research. However, it is susceptible to salinity, with most cultivated varieties showing reduced growth at salt levels above 3 dS/m. Despite numerous efforts to improve its salt tolerance, little progress has been made. A promising area of research lies in the study of epigenetic regulation, which encompasses DNA methylation, histone modifications, and chromatin remodelling. These processes play a crucial role in mediating how plants respond to salt stress by modulating gene expression. This often results in heritable changes that can be used as molecular markers. Studies in rice and other cereals have demonstrated a clear association between histone alterations, shifts in DNA methylation patterns, and the expression of salt-responsive genes. Furthermore, epigenetic mechanisms contribute to the development of stress memory, enabling plants to respond more effectively to recurring stressful conditions. Understanding these regulatory pathways offers new opportunities for breeding or engineering salt-tolerant rice varieties, potentially leading to improved crop resilience and productivity under saline conditions.

## 1. Introduction

Salinity refers to the accumulation of high concentrations of soluble salts, such as sodium chloride, magnesium, calcium sulfates, and bicarbonates, in soil and water. Globally, recent assessments show that nearly 1.38 billion hectares of land (over 10% of total land area) are currently affected by salinity, with another 1 billion hectares at risk due to climate change and human mismanagement [1]. In terms of soil layers, approximately 424 million hectares of topsoil (0–30 cm) and 833 million hectares of subsoil (30–100 cm) are saline or sodic [2].

Each year, an additional up to 2 million hectares become too saline for cultivation, contributing to a steadily growing global crisis [3]. While some salinity arises from natural processes, human activities and climate change are amplifying the issue. Examples include irrigation with inadequate drainage, deforestation that reduces evapotranspiration and increases groundwater recharge (thereby raising water tables), and climate-driven factors such as sea-level rise, erratic precipitation, higher evaporation rates, and permafrost thawing [1,3]. Natural processes, including the deposition of oceanic salts through rainfall, aeolian transport, and the weathering of parent rocks, contribute to primary salinisation. In contrast, human mismanagement, such as irrigation with saline water, groundwater overextraction, and excessive fertiliser application, drives secondary salinisation [4].

Of the world’s irrigated lands, about 33% are already salinity-affected, a sharp increase from older estimates (which suggested around 20%) [5]. Importantly, climate change does not act uniformly across the globe: projections indicate significant increases in salinity in drylands of South America, southern Australia, Mexico, and South Africa, while some regions, such as Eastern Europe, Turkmenistan, and the Horn of Africa, may experience stable or even decreasing salinity levels depending on local hydrological conditions and emission pathways [4].

Crops grown in saline environments face multiple challenges, including limited water availability due to osmotic stress, toxic ion build-up, nutrient imbalances, and deteriorating soil structure, all of which reduce agricultural productivity [6,7]. Recent research has revealed that plant responses to salinity are shaped not only by genetic variation but also by epigenetic mechanisms. DNA methylation changes are among the most consistent responses to salt stress, reprogramming the expression of stress-responsive genes and, in some cases, contributing to stress memory [8,9]. Histone modifications such as H3K4me3, H3K9ac, and H3K27me3 are dynamically redistributed under salinity, altering chromatin accessibility and transcriptional activity [10,11]. Likewise, small RNAs play crucial roles by targeting transcription factors, ion transporters, and signaling components that modulate salt tolerance [12]. Importantly, several studies emphasize that these epigenetic changes can persist across developmental stages, creating a molecular basis for stress memory that enables more efficient responses to recurring salt exposure [11,13]. Moreover, there is growing evidence that epigenetic marks and small RNA pathways can be transmitted across generations, providing a mechanism for transgenerational adaptation to saline environments [14,15].

These epigenetic changes can be triggered by salt stress, persist across developmental stages, and in some cases be inherited, creating a “stress memory” that influences plant performance under recurring stress [8,9,11,15,16].

With the global population projected to reach 9.6 billion by 2050, food production must rise by about 70% (roughly 44 million tonnes annually) to meet demand [17,18,19]. This task is made more difficult by the scarcity of new arable land and the increasing severity of environmental stresses [20,21,22]. Among these, soil salinity and water scarcity are considered the two most pressing constraints on global crop yields [23]. In light of these challenges, developing crops that can thrive in salt-affected soils is essential for future food security.

Rice is a staple food for more than half of the world’s population and belongs to the genus *Oryza* within the grass family Poaceae. Among the many species in this genus, only two are cultivated: *Oryza sativa* L., which is widely grown throughout Asia, and *Oryza glaberrima* Steud., which originated in and is mainly cultivated in West and Central Africa [24,25]. Today, rice is cultivated in 114 countries across six continents [26]. In the humid and sub-humid regions of Asia, it is the most important agricultural commodity, providing a primary source of employment and income. For more than three billion people in Asia, rice supplies between 50% and 80% of their daily caloric intake [27,28].

Despite its importance, rice is highly vulnerable to salt stress, making it the most salt-sensitive cereal crop. Most widely cultivated rice varieties have a salinity threshold of about 3 dS/m [29], while soils are generally classified as saline when their electrical conductivity (ECe) exceeds 4 dS/m [30]. Yield losses occur even under moderate salinity; for example, an ECe of 3.5 dS/m can cause a 10% yield reduction, and a level of 7.2 dS/m may reduce yields by 50% [4]. Although Asia accounts for 90% of global rice production and consumption [17,18,19], salinity stress is becoming an increasingly severe challenge. Rising sea levels are intensifying salinity in major Asian river deltas, which represent some of the world’s most fertile rice-growing areas [31]. Moreover, abiotic stresses resulting from anthropogenic activities, such as heat stress, drought stress, and heavy metal stress, are well-documented to disrupt the molecular networks that maintain root meristem integrity, affecting auxin transport, cytokinin cross-talk, and microtubule organisation [32,33,34]. Emerging evidence also suggests that epigenetic regulation modulates these pathways, influencing how rice perceives and responds to combined abiotic stresses. Understanding these epigenetic mechanisms offers new avenues for developing rice varieties with enhanced and heritable tolerance to salinity [35,36,37,38].

Given these challenges, enhancing rice tolerance to salinity stress is crucial for sustaining food security in regions that rely heavily on this staple crop. Although progress has been made in developing salt-tolerant rice varieties, overall advancements remain limited. This review therefore emphasizes not only physiological and genetic approaches but also epigenetic strategies for improving rice salinity tolerance, including the roles of DNA methylation, histone modifications, and small RNA pathways in adaptive stress responses.

## 2. Salt Stress Responses and Adaptive Mechanisms in Rice

Plants are generally classified into two groups based on their tolerance to salinity: halophytes and glycophytes. Halophytes can withstand high salt concentrations, up to 400 mM NaCl, whereas glycophytes, which include most major crops, perform poorly under saline conditions [39]. Among cereals, rye (*Secale cereale*) exhibits relatively high salt tolerance, with a threshold of 11 dS/m, while rice is the most sensitive, showing stress symptoms at levels as low as 3 dS/m [29].

**Figure 1:**
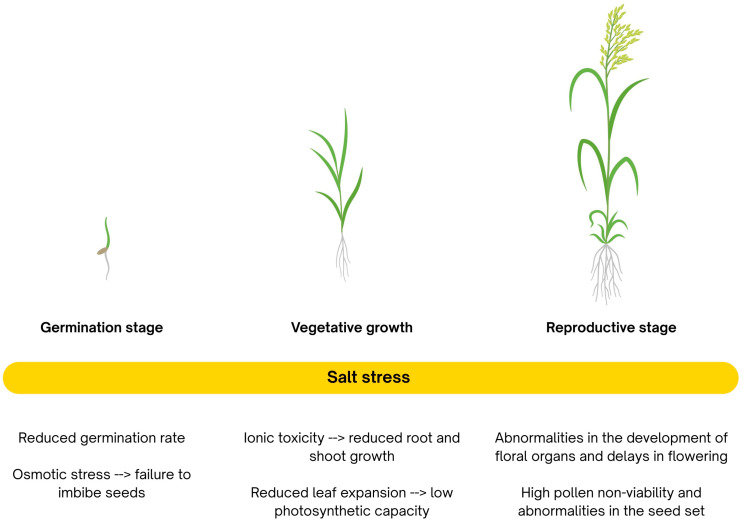
Stage-specific effects of salt stress on rice development. Salt stress adversely affects rice growth and productivity across all major phenological stages. During the germination stage, osmotic stress inhibits water uptake, leading to reduced germination rates or failure of seed imbibition. In the vegetative growth phase, ionic toxicity restricts root and shoot elongation, reduces leaf expansion, and lowers photosynthetic capacity. At the reproductive stage, salt stress induces floral abnormalities, delays flowering, and causes high pollen non-viability and defective seed set, collectively reducing grain yield.

Research indicates that rice demonstrates some tolerance to salinity during certain growth stages, such as germination, active tillering, and late maturity. However, it is especially vulnerable during early seedling development and the reproductive phase [40,41]. Even relatively low salt concentrations, around 50 mM NaCl, can cause significant yield losses in rice (Figure 1) [42]. Emerging studies show that these stage-specific responses are influenced not only by genetic factors but also by epigenetic regulation, such as DNA methylation and small RNA-mediated modulation, which can alter stress-responsive gene expression during critical developmental windows [8,35,43,44,45,46].

**Figure 2:**
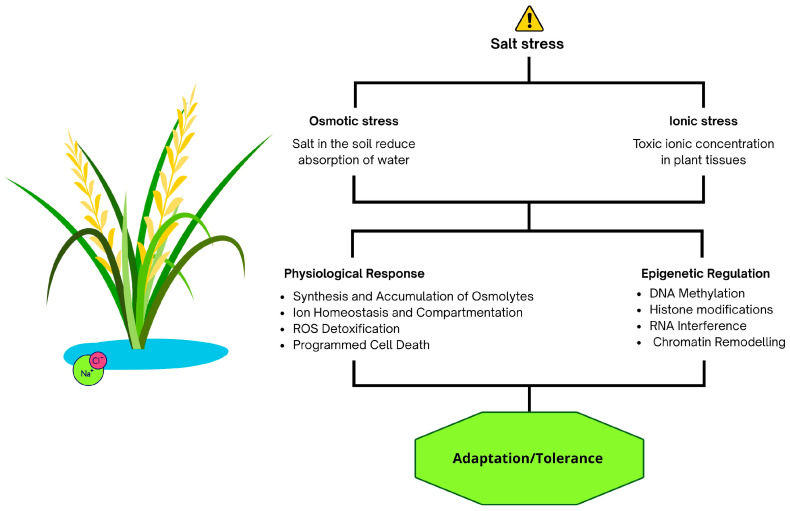
Overview of rice responses to salt stress at the physiological and epigenetic level. Salt stress in rice induces osmotic stress, caused by reduced water uptake, and ionic stress, resulting from toxic ion accumulation. Physiological responses include osmolyte synthesis, ion homeostasis via compartmentalization, ROS detoxification, and programmed cell death under severe stress. In parallel, epigenetic regulation—via DNA methylation, histone modifications, RNAi, and chromatin remodeling—fine-tunes gene expression, coordinating these stress responses and enhancing adaptive tolerance.

Salinity affects plants through two interconnected mechanisms: osmotic stress and ionic stress (Figure 2). Osmotic stress limits water uptake, reducing hydration and growth, while ionic stress results from the toxic accumulation of ions (e.g., Na^+^, Cl^−^) in plant tissues, particularly in older leaves. Excess Na^+^ in the cytoplasm and vacuoles disrupts cellular processes, potentially leading to metabolic dysfunction and programmed cell death.

Recent reviews highlight how DNA methylation, histone modifications, and non-coding RNAs modulate the expression of ion transporters, enzymes for osmolyte biosynthesis, and antioxidant systems [16,47,48]. For instance, transporters of the HKT1 family, which are essential for maintaining sodium/potassium homeostasis, are under epigenetic regulation, thereby contributing to the control of toxic ion accumulation in plant tissues [49]. Similarly, changes in DNA methylation influence the expression of genes responsible for proline biosynthesis, promoting the accumulation of this key osmoprotectant under salt stress [50]. Moreover, emerging evidence emphasizes the interplay between redox status and epigenetic regulation, with reactive oxygen species affecting both DNA methylation and histone acetylation, ultimately modulating the activation of antioxidant genes [51]. As in other plant species, rice has evolved several adaptive mechanisms to mitigate the effects of salinity stress. These include: (i) accumulation of osmolytes; (ii) maintenance of ion homeostasis through compartmental sequestration; (iii) activation of antioxidant systems against reactive oxygen species (ROS); and (iv) initiation of programmed cell death to limit cellular damage.

Like many plant species, rice has developed multiple adaptive strategies to cope with salinity stress. These include (i) the synthesis and accumulation of osmoprotective compounds (osmolytes); (ii) regulation of ion homeostasis and sequestration within specific cellular compartments; (iii) activation of antioxidant systems to detoxify reactive oxygen species (ROS); and (iv) initiation of programmed cell death as a controlled mechanism to restrict cellular damage. Importantly, these physiological responses are fine-tuned by epigenetic mechanisms, DNA methylation, histone modifications, chromatin remodelling, and RNA interference (RNAi), which dynamically regulate stress-responsive genes, enabling rice to adapt to fluctuating salinity levels and maintain homeostasis under adverse conditions.

### 2.1. Organic Osmolytes and Their Role in Rice Salinity Tolerance

Organic osmolytes are small solute molecules that help cells regulate internal water balance, particularly under conditions of osmotic stress. These compounds can be grouped into four major categories: (i) small carbohydrates such as trehalose, and polyols including glycerol, sorbitol, and inositols; (ii) amino acids and their derivatives, such as proline, glycine, and taurine; (iii) methylated compounds, including glycine betaine and dimethyl-sulphoniopropionate (DMSP); and (iv) urea [52]. Depending on external osmotic conditions, plants can synthesise or degrade these osmolytes, enabling cells to prevent excessive water loss and shrinkage in volume [52]. The early phase of salinity stress is characterised by osmotic imbalance, caused by high external salt concentrations that restrict water uptake and immediately inhibit plant growth [7,53]. Under normal conditions, plant cells absorb water and nutrients from the surrounding soil, maintaining higher internal osmotic pressure. However, when soil salinity rises, the osmotic potential outside the roots exceeds that inside the plant cells, thereby reducing their capacity to absorb water and minerals [54]. To counteract this, plants engage in osmotic adjustment by accumulating compatible solutes, organic osmolytes that decrease cellular osmotic potential and facilitate sustained water uptake [55,56]. Recent studies indicate that osmolyte accumulation is also influenced by epigenetic regulation. In wheat and rice, DNA methylation at the promoters of key biosynthetic genes such as P5CS (for proline synthesis) and BADH (for glycine betaine) was shown to modulate their transcription, thereby influencing osmolyte accumulation and stress tolerance under drought and osmotic stress conditions [50,57]. Small RNAs also play a pivotal role: in cotton, specific microRNAs directly regulate P5CS1, affecting proline biosynthesis under salt stress [58]. In addition, histone modifications shape the transcriptional response to abscisic acid (ABA), a central hormone in osmotic stress signaling, indicating that chromatin remodelling contributes to the regulation of stress-responsive gene networks [59]. Together, these studies provide clear evidence that DNA methylation, histone modifications, and small RNA pathways converge to regulate osmotic adjustment and stress signaling in plants.

In rice, the accumulation of specific osmolytes has been shown to enhance salt tolerance. Trehalose, for instance, accumulates in rice under saline conditions. At low to moderate concentrations, it reduces Na^+^ uptake, downregulates stress-responsive genes such as *salT*, and alleviates growth inhibition. At higher concentrations, trehalose helps maintain chlorophyll content, protect root structures, and sustain overall plant growth under salinity stress [60]. Epigenetic modifications, including histone acetylation and DNA methylation, can influence trehalose biosynthetic gene expression, providing a flexible, environment-responsive mechanism for osmotic adjustment. Transgenic crops overexpressing trehalose biosynthetic genes (*TPS* and *TPP*) exhibit increased tolerance to salt, drought, and cold stress, likely through membrane stabilization, reduced ion leakage, and decreased lipid peroxidation [61,62].

Proline is another key osmolyte that accumulates in response to salinity. Salt-tolerant rice cultivars generally produce more proline than sensitive ones under stress conditions [63,64]. Proline functions both as an osmoprotectant and antioxidant, stabilizing proteins and cellular structures, scavenging reactive oxygen species, and maintaining cytosolic redox balance [65,66]. Its biosynthesis mainly proceeds via the glutamate pathway, involving pyrroline-5-carboxylate synthase (P5CS) and reductase (P5CR), whose transcription is subject to epigenetic regulation, including promoter methylation and histone modifications, affecting proline accumulation under stress. In rice, and other crop species, osmotic stress induces changes in DNA methylation patterns that correlate with altered expression of *P5CS* and *P5CR*, ultimately contributing to enhanced proline accumulation; these methylation marks can even be transmitted across generations, suggesting a role in stress memory [50,67,68]. Similarly, in wheat, drought priming was shown to reduce promoter methylation of *TaP5CS*, thereby facilitating its transcriptional activation and increased proline levels under subsequent stress [8]. In *Arabidopsis*, histone modifications also participate in this regulation: salt stress-induced transcriptional memory of *P5CS1* is maintained by enrichment of the active mark H3K4me3 at its promoter, leading to faster and stronger induction of proline biosynthesis during recurrent dehydration [69].

Glycine betaine, a naturally occurring quaternary ammonium compound present in many organisms [70], is widely recognised as an effective osmoprotectant [71,72]. Although rice does not naturally synthesize glycine betaine because it lacks key enzymes in the biosynthetic pathway, such as choline monooxygenase and betaine aldehyde dehydrogenase, studies have demonstrated that rice can still benefit from its external application.

Research by Harinasut et al. (1996) demonstrated that exogenously applied glycine betaine can be absorbed by rice and accumulate in the leaves, where it enhances photosynthetic efficiency by maintaining the quantum yield of photosystem II under salt stress [73]. In *japonica* varieties such as cv. Nipponbare, some activity of betaine aldehyde dehydrogenase (BADH), the enzyme responsible for converting betaine aldehyde into glycine betaine in the biosynthetic pathway, has been detected [74]. This finding suggests that rice has a limited but functional capacity for endogenous glycine betaine production, which could contribute to stress tolerance.

Myo-inositol and its derivatives also act as important osmoprotectants in rice. Myo-inositol serves as a precursor for a range of stress-associated metabolites, including raffinose family oligosaccharides (RFOs). Its accumulation supports osmotic balance and protects photosynthetic machinery under stress conditions. For instance, overexpression of the *PINO1* gene from the halophyte *Porteresia coarctata* in transgenic tobacco enhanced salt tolerance by regulating ion homeostasis and maintaining photosynthetic efficiency under saline conditions [75]. *PINO1* encodes a protein involved in controlling sodium and potassium transport, helping plants limit toxic ion accumulation and sustain growth during salt stress.

### 2.2. Ionic Homeostasis and Ion Transport Mechanisms in Rice Under Salinity Stress

Under saline conditions, elevated sodium ion (Na^+^) levels in the external environment create a steep electrochemical gradient across the plasma membrane. This gradient drives the passive entry of Na^+^ into plant cells, often through potassium (K^+^) transport channels, resulting in an increased cytoplasmic Na^+^ concentration [76]. Excess intracellular Na^+^ interferes with cellular metabolism by competing with K^+^, an essential ion for activating enzymes and supporting protein synthesis [76,77]. Recent evidence indicates that the expression and activity of ion transporters are also modulated by epigenetic mechanisms, including DNA methylation, histone modifications, and small RNAs, allowing plants to fine-tune Na^+^/K^+^ homeostasis under fluctuating salinity. For instance, DNA methylation patterns have been shown to directly influence the activity of HKT1;5, a key Na^+^ transporter responsible for maintaining Na^+^/K^+^ homeostasis in rice, by altering promoter accessibility and the interaction with transposable elements [47]. More broadly, DNA methylation, along with histone modifications and non-coding RNAs, constitutes an adaptive layer of regulation that allows plants to fine-tune ion homeostasis and physiological responses under fluctuating salinity [16,78].

To cope with salinity, plants employ multiple strategies to limit Na^+^ accumulation, particularly in sensitive tissues. A primary mechanism is the restriction of Na^+^ uptake at the root level, which helps maintain lower Na^+^ concentrations in aerial parts, especially leaves. When this exclusion mechanism fails, the toxic effects of Na^+^ accumulation may not be immediately visible but eventually lead to premature ageing and death of older leaves [6]. The concentration of Na^+^ in leaf tissue is strongly associated with salinity tolerance in rice, with this relationship observed in both indica and japonica subspecies [79]. Epigenetic regulation can influence the transcriptional responsiveness of key Na^+^ transport genes in different tissues, contributing to genotype-specific tolerance patterns [37,48,80].

Maintaining a low cytosolic Na^+^/K^+^ ratio is critical for sustaining ionic homeostasis under stress. This ratio directly influences metabolic stability, photosynthetic efficiency, and overall plant resilience [81,82,83]. Transporter proteins such as OsHKT1;5, OsHAK10, and OsHAK16 play key roles in regulating long-distance Na^+^ transport. Their expression is often localized to older leaves, suggesting a mechanism for redistributing Na^+^ to minimize toxicity in younger tissues [84]. Histone acetylation and promoter methylation of these transporters can alter their expression under stress, providing an additional layer of adaptive control.

Another important mechanism is vacuolar sequestration, particularly in salt-tolerant genotypes such as Pokkali. In these plants, Na^+^ is rapidly transported into vacuoles by tonoplast-localised Na^+^/H^+^ antiporters (OsNHX1–4), thereby reducing cytosolic Na^+^ levels and limiting its interference with essential cellular functions. Overexpression of *OsNHX1* has been successfully implemented in both rice and maize (*Zea mays*), leading to improved salt tolerance in transgenic lines of both species. In rice, constitutive overexpression of *OsNHX1* has been shown to enhance salinity tolerance by increasing the vacuolar sequestration of sodium ions, thereby reducing their toxic effects on cellular metabolism. Similarly, in maize, transgenic plants expressing *OsNHX1* exhibited increased biomass accumulation and higher grain yields under saline conditions, demonstrating the gene’s potential to confer salt tolerance across different plant species.

These findings underscore the functional conservation of *OsNHX1* across species and its potential utility in developing salt-tolerant crops through genetic engineering [85,86]. Epigenetic modifications, including DNA methylation and histone acetylation at OsNHX loci, may influence the efficiency of vacuolar sequestration and the stability of tolerance traits [16,36].

Plasma membrane Na^+^/H^+^ antiporters, such as OsSOS1, contribute to cytosolic Na^+^ extrusion. Their activity is regulated by the SOS signaling pathway, which involves the calcium sensor OsCBL4 and the protein kinase OsCIPK24, forming a functional module that enhances salt tolerance by activating OsSOS1 and promoting Na^+^ efflux [86,87]. Interestingly, truncated variants of OsSOS1 lacking the autoinhibitory domain display stronger Na^+^ transport activity, highlighting their potential for bioengineering applications [87].

In addition to Na^+^ transport, maintaining K^+^ uptake is equally critical. Inward-rectifying K^+^ channels (Kir channels), such as OsAKT1, mediate selective K^+^ influx under normal conditions. However, salt stress suppresses OsAKT1 expression, thereby reducing K^+^ acquisition and aggravating ionic imbalance [88]. Notably, overexpression of the transcription factor OrbHLH001 restores OsAKT1 expression and improves salt tolerance in rice [89]. Small RNAs and histone modifications may further fine-tune K^+^ channel activity, enabling dynamic adjustment to saline environments [90].

Vacuolar and endosomal H^+^-ATPases also play a central role by generating the proton gradient required for secondary active transport via Na^+^/H^+^ antiporters. The gene OSA3, which encodes a vacuolar H^+^-ATPase, is strongly induced in salt-tolerant mutants but not in sensitive cultivars, underscoring its importance in the salinity response [86].

Moreover, chloride channel genes such as OsCLC1 show differential regulation between salt-tolerant and salt-sensitive rice varieties. In the sensitive cultivar IR29, OsCLC1 expression is downregulated under salt stress, whereas in the tolerant variety Pokkali, it is upregulated. This suggests a potential role in Cl^−^ compartmentation and ionic detoxification [91]. A summary of the major players in this regulatory network is presented in Table 1.

### 2.3. ROS Accumulation and Antioxidant Responses in Rice Under Salt Stress

Reactive oxygen species (ROS) are highly reactive molecules that play dual roles in plants: at controlled levels, they act as essential signaling molecules regulating growth, development, and stress responses, but when overproduced, they cause oxidative damage to proteins, lipids, and nucleic acids [93,94,95]. Salinity stress often induces excessive ROS generation, which can overwhelm cellular antioxidant defenses, leading to oxidative stress and, in severe cases, cell death [96,97,98]. Recent evidence suggests that the regulation of ROS homeostasis extends beyond classical transcriptional and biochemical control, involving epigenetic mechanisms that allow plants to dynamically adapt to stress conditions. Shriti et al. (2024) demonstrated that stress-induced ROS accumulation can trigger changes in DNA methylation at stress-responsive loci, which in turn reshapes gene expression patterns related to antioxidant defense [99]. This supports the view that ROS not only act as damaging agents but also as signaling molecules that directly influence chromatin states. In line with this, Kaya and Adamakis (2025) highlighted how redox–epigenetic crosstalk modulates the activity of DNA methyltransferases and histone-modifying enzymes, thereby providing plants with an additional layer of regulatory flexibility [51]. Taken together, these findings indicate that ROS-mediated epigenetic modifications contribute to the fine-tuning of defense pathways, enabling a more nuanced and reversible adjustment of stress responses compared to purely transcriptional regulation.

To mitigate ROS toxicity, plants depend on a tightly regulated antioxidant defence system that includes both enzymatic and non-enzymatic components [100,101,102,103]. Enzymatic antioxidants comprise superoxide dismutase (SOD), which catalyses the conversion of superoxide radicals into H_2_O_2_; catalase (CAT), which further decomposes H_2_O_2_ into water and oxygen; and enzymes such as ascorbate peroxidase (APX) and glutathione reductase (GR), which function within the ascorbate–glutathione cycle to sustain redox homeostasis [101,102]. Non-enzymatic antioxidants consist of small molecules such as ascorbate (ASC), glutathione (GSH), carotenoids, and tocopherols, which directly neutralise ROS and contribute to cellular protection under stress. For instance, salt-tolerant rice varieties such as Pokkali display higher activity of detoxifying enzymes like CAT and greater accumulation of antioxidant molecules, including ASC and GSH, compared to sensitive varieties like Pusa Basmati. Such coordinated responses allow tolerant cultivars to minimise oxidative damage, maintain membrane integrity, and preserve photosynthetic efficiency under high salinity stress [95]. Dionisio-Sese et al. [104] demonstrated that salt-tolerant varieties sustain higher activities of these antioxidant enzymes under salinity, thereby mitigating cellular damage and enhancing stress resilience. Their findings also indicated that the imbalance between ROS production and detoxification capacity in sensitive cultivars contributes to their susceptibility to salinity-induced oxidative injury. It is now thought that epigenetic differences between tolerant and sensitive genotypes may underlie this divergence, with more stable antioxidant gene expression profiles in tolerant lines.

### 2.4. Salinity-Induced Programmed Cell Death: Mechanisms and Roles in Rice

Plants are sessile organisms and are therefore continuously exposed to various stressors, including high soil salinity and drought. Although they can activate multiple protective responses, excessive or prolonged saline stress may trigger programmed cell death (PCD), a genetically regulated process that removes damaged or non-functional cells to safeguard overall plant health [105,106]. PCD is an evolutionarily conserved mechanism across multicellular life, essential for maintaining tissue integrity, guiding development, and eliminating superfluous or injured cells during abiotic stress [107,108,109]. Recent studies show that programmed cell death (PCD) is controlled not only genetically but also epigenetically. DNA methylation, histone modifications, and chromatin remodeling regulate the expression of PCD-related genes, particularly under stress. Evidence from plants indicates that such epigenetic changes influence chromatin accessibility and gene activation during abiotic and hypoxic stress, underscoring the central role of chromatin dynamics in PCD [110,111].

In rice, salinity-induced PCD occurs mainly in root tissues, functioning as a localised containment mechanism. Liu et al. (2007) showed that NaCl-treated rice roots display a spatially organized pattern of cell death, particularly in cortical cells, which likely serves as a barrier to restrict Na^+^ influx into the stele and thereby reduce its accumulation in the shoot [112]. Further evidence indicates that PCD also takes place in the root apical meristem under salt stress, suggesting a targeted strategy to confine and minimise broader damage [113].

Further molecular evidence supports the role of programmed cell death (PCD) in salinity responses. Proteomic and transcriptomic analyses of rice roots under salt stress revealed upregulation of PCD-associated markers, including caspase-like proteases, metacaspases, and vacuolar processing enzymes such as OsVPE3. Notably, Lu et al. (2016) demonstrated that suppression of OsVPE3 delays vacuolar membrane rupture, reducing PCD and thereby enhancing salt tolerance in rice [114]. Moreover, mitochondrial dysfunction and cytochrome c release, early hallmarks of PCD, have been observed within hours of salt treatment, alongside transient bursts of ROS and initial increases in antioxidant enzyme activities. Yet, prolonged exposure leads to impaired detoxification capacity and progression of PCD in root tip cells, as documented by Li et al. (2007) and Chen et al. (2009) [113,115]. Emerging evidence indicates that epigenetic modifications such as stress-induced histone acetylation and DNA demethylation may influence the transcription of PCD regulators like metacaspases and vacuolar processing enzymes, thereby fine-tuning cell death decisions under salt stress [116,117]. In other plant species, environmental stresses have been shown to modulate the expression of genes involved in defense and development. For example, in *Citrus limon*, exposure to stress conditions upregulates the germin-like protein gene ClGLP1, which is implicated in mitigating oxidative stress and defending against pathogens [118]. These observations align with studies indicating that controlled PCD helps prevent uncontrolled cell lysis and ROS spillover, thereby protecting surrounding tissues from secondary damage [86].

## 3. Epigenetic Mechanisms and Stress Memory in Rice Under Salt Stress

Among the molecular mechanisms underlying stress adaptation in rice, epigenetic regulation plays a pivotal role. Epigenetic changes alter gene expression without modifying the DNA sequence, enabling plants to adjust to environmental challenges rapidly. Importantly, some of these modifications can be stably maintained and transmitted across generations, forming a “stress memory” that enhances resilience in progeny.

Key epigenetic mechanisms include

**Figure 3:**
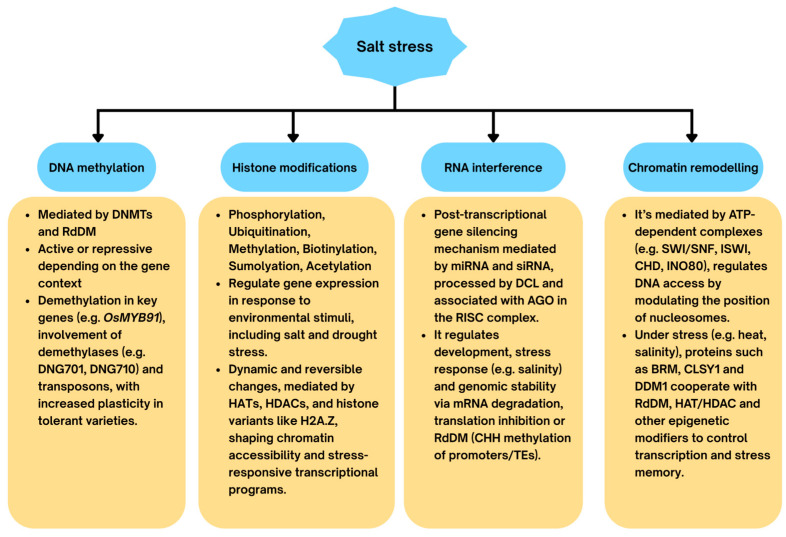
Epigenetic mechanisms underlying rice response to salinity. Under salt stress, rice plants employ epigenetic mechanisms that fine-tune gene expression through dynamic and reversible modifications. DNA methylation, mediated by DNA methyltransferases (DNMTs) and the RdDM pathway, can function as either a repressive or activating mark depending on genomic context. Demethylation of stress-related genes and activation of transposable elements have been linked to enhanced tolerance in certain varieties. Histone modifications (e.g., acetylation, methylation) alter chromatin structure and accessibility, influencing transcription of stress-responsive genes. RNA interference (RNAi), involving miRNAs and siRNAs, regulates gene expression post-transcriptionally via mRNA cleavage, translation inhibition, or reinforcement of RdDM. Chromatin remodelling complexes (e.g., SWI/SNF, ISWI) reposition nucleosomes, facilitating access to stress-inducible promoters. Collectively, these interconnected epigenetic layers contribute to the plasticity of the rice epigenome and enhance tolerance to salinity stress.

- DNA methylation, primarily at cytosine residues.
- Histone modifications, such as acetylation, methylation, phosphorylation, ubiquitination, glycosylation, and sumoylation.
- Histone variants, which replace canonical histones and modify nucleosome composition.
- Non-coding RNAs (ncRNAs) that fine-tune transcriptional activity.
- Chromatin remodelling, which regulates nucleosome density and DNA accessibility (Figure 3).

These mechanisms operate individually or in combination, creating a dynamic epigenetic landscape that regulates the expression of stress-responsive genes. The functional outcome of an epigenetic modification depends not only on its type but also on its genomic context—for example, whether it occurs near coding regions or transposable elements [119].

In addition, RNA interference (RNAi) pathways, particularly those involving small RNAs (sRNAs), reinforce epigenetic regulation by guiding sequence-specific DNA methylation and histone modifications [120].

Stress memory in plants refers to the ability to “remember” prior exposure to environmental stress and respond more effectively to subsequent challenges. This phenomenon can occur within the same generation (somatic memory) or be passed to offspring (transgenerational memory). Mechanistically, stress memory is maintained through stable epigenetic marks—such as persistent DNA methylation at stress-inducible promoters, or histone modifications that keep chromatin in a more accessible state. These marks prime the transcriptional machinery, allowing faster or stronger reactivation of protective genes upon re-exposure to stress. In rice and other cereals, stress memory contributes to enhanced tolerance to salinity, drought, and temperature fluctuations, making it a critical adaptive strategy in fluctuating environments.

Recent studies in cereals, including rice, demonstrate that natural or induced epigenetic variation can significantly enhance tolerance to salinity. This opens opportunities for crop improvement through approaches such as identifying beneficial epigenetic variants or applying targeted interventions to induce favourable epigenetic states. Consequently, epigenetic research and tools represent a promising frontier in developing salt-tolerant rice cultivars [121,122,123,124,125].

### 3.1. DNA Methylation Dynamics and Epigenetic Regulation of Salinity Stress Responses in Rice

DNA methylation is a central epigenetic mechanism regulating plant responses to salinity, influencing both stress adaptability and cultivar-specific tolerance. In plants, cytosine methylation occurs in CG, CHG, and CHH contexts, catalysed by DNA methyltransferases (DNMTs), while DNA glycosylases mediate active demethylation. Beyond its classical role in gene silencing, DNA methylation contributes to stress memory and adaptive plasticity by dynamically shaping transcriptional programs in response to environmental stress [126,127,128,129,130,131,132,133].

Under salt stress, rice exhibits dynamic and context-dependent methylation changes. Promoter methylation typically represses gene expression; however, stress can induce both hypermethylation and hypomethylation at specific loci, thereby fine-tuning transcriptional activity. Notably, CHH methylation has been linked to the activation of stress-responsive genes [134], while demethylation at key loci such as the *OsMYB91* promoter enhances transcriptional responsiveness [135,136]. These patterns highlight the flexible regulatory role of DNA methylation rather than a uniform gene-silencing mechanism.

Cultivar-specific differences provide critical insights into tolerance mechanisms. Comparative studies of the salt-tolerant variety Pokkali and the sensitive IR29 revealed striking contrasts: Pokkali exhibited a pronounced global decline in 5mC levels under salinity, accompanied by strong induction of demethylases (DNG701, DNG710), suggesting a rapid demethylation-driven transcriptional reprogramming. In contrast, IR29 displayed modest and statistically insignificant methylation changes, potentially reflecting reduced methylome plasticity [137].

Demethylation in Pokkali extended to transposable elements (TEs) and repetitive sequences, including evidence of stress-induced TE activation responses absent in IR29. Nuclear reorganisation of methylated regions in Pokkali roots further underscores the structural dimension of methylome remodelling [137].

Functional studies reinforce these observations. Rice mutants deficient in *OsDRM2* (a de novo DNMT) exhibited impaired seed germination and reduced root biomass under salinity, implicating locus-specific methylation in stress resilience. Similarly, mutations in the histone acetyltransferase *oshac704* altered DNA methylation, demonstrating crosstalk between histone modifications and methylation pathways. Interestingly, both mutants displayed greater salt-induced demethylation yet maintained moderate stress tolerance, suggesting that epigenetic flexibility itself may confer adaptive benefits [137].

Taken together, these findings demonstrate that the capacity to reconfigure methylation, rather than maintaining static patterns dynamically, underlies salt tolerance in rice. Cultivar-specific contrasts, particularly between Pokkali and IR29, highlight methylome plasticity as a determinant of resilience. While some methylation marks contribute to transgenerational stress memory, only a fraction is stably inherited, limiting their predictive value for gene expression in progeny [138,139]. Nonetheless, overexpression of demethylases has been linked to enhanced tolerance [140], underscoring the potential of manipulating DNA methylation for crop improvement (Figure 3).

### 3.2. Histone Modifications as Epigenetic Regulators of Salinity Stress Responses in Plants

Histone modifications represent a major epigenetic mechanism that regulates chromatin structure and transcriptional activity [141], playing a critical role in plant adaptation to salinity. These modifications occur primarily on histone N-terminal tails and include acetylation, methylation, phosphorylation, ubiquitination, and other chemical marks. By altering DNA–histone interactions or recruiting regulatory proteins, they can either activate or repress stress-responsive gene expression [142,143]. In addition, histone variants such as H2A.Z contribute to chromatin remodelling, linking structural flexibility with stress perception [144,145,146].

A key feature of histone modifications is their dynamic and reversible nature, mediated by “writers” (enzymes that deposit marks), “erasers” (which remove them), and “readers” (which interpret them) [147]. This reversibility enables plants to fine-tune transcription in response to fluctuating environmental conditions, including salinity, and to integrate stress signaling with developmental programs.

Histone acetylation emerges as a central regulator of rapid stress responses. Marks such as H3K9ac and H3K27ac are associated with open chromatin and transcriptional activation, and their enrichment has been documented at promoters of salt-responsive genes in rice, maize, and wheat [148,149,150]. Acetylation is mediated by histone acetyltransferases (HATs) and reversed by histone deacetylases (HDACs). For example, in rice, HAC1 and related HAT genes enhance drought and salt tolerance via increased acetylation of H3 [151,152]. Similarly, maize HATs (ZmGCN5, ZmHATB) promote hyperacetylation of stress-responsive promoters, while in *Arabidopsis*, GCN5 activates cell wall–modifying genes linked to salt tolerance [153,154]. HDACs also play important roles in fine-tuning responses: rice HDT701 enhances seedling tolerance, while HDA9 reduces sensitivity to drought and salinity by modulating H3K9 acetylation levels [155,156]. In wheat, TaHDA4 interacts with TaHOS15 to repress defense-related genes, ensuring balanced responses [157,158].

Histone methylation, in contrast, is generally associated with longer-term regulatory processes such as developmental transitions and stress memory. Stable repressive marks like H3K27me3, deposited by the Polycomb Repressive Complex 2 (PRC2), silence developmental and stress-related genes, providing a form of transcriptional memory that can persist across cell divisions [159,160]. This stability distinguishes methylation from acetylation and links it to epigenetic priming and the adaptation to transgenerational stress.

Importantly, histone modifications do not act in isolation but show crosstalk with other epigenetic processes. For instance, histone acetylation can enhance the accessibility of DNA regions that remain methylated, partially overriding transcriptional repression [161]. Stress-responsive transcription factors also serve as recruiters for histone-modifying complexes: rice IDS1 and *Arabidopsis* MYB96 interact with histone modifiers to activate salinity responses, while OsbZIP46 coordinates H2B ubiquitination during drought [147,162]. These examples underscore the tight integration between signaling pathways and chromatin dynamics.

In summary, histone modifications provide a multilayered regulatory system that enables plants to mount both rapid and sustained responses to salinity. Acetylation supports rapid transcriptional reprogramming, while methylation underlies longer-term memory. Their interaction with transcription factors and DNA methylation ensures precise control of stress-responsive networks. Together, these mechanisms underscore the potential of targeting histone modifiers in crop improvement strategies to enhance salinity tolerance. Figure 3 illustrates key histone modification pathways and their roles in salt stress adaptation.

### 3.3. RNA-Mediated Epigenetic Regulation and Post-Transcriptional Gene Silencing in Plants

Epigenetic regulation in plants extends beyond transcriptional control to include post-transcriptional and translational regulation, primarily through the degradation of target mRNAs or inhibition of their translation. This process, broadly termed RNA interference (RNAi), provides precise temporal and spatial control over gene expression, thereby fine-tuning growth, development, and stress responses (Figure 3) [163].

At the core of RNAi are small non-coding RNAs (sRNAs), which are broadly classified into microRNAs (miRNAs) and small interfering RNAs (siRNAs) [164]. Both classes share conserved biogenetic pathways: they are processed from double-stranded RNA precursors by Dicer-like (DCL) enzymes, then loaded into ARGONAUTE (AGO)-containing RNA-induced silencing complexes (RISC). The RISC complex recognizes complementary mRNAs through the AGO PAZ domain and mediates silencing via translation inhibition, mRNA cleavage, or recruitment of chromatin modifiers [165,166,167,168,169,170].

miRNAs are key regulators of plant stress responses, development, and genome stability [171]. By downregulating specific targets, miRNAs ensure appropriate physiological responses. Under salinity stress, miRNAs may either reduce tolerance (by repressing protective genes) or enhance resilience (when their suppression allows stress-responsive genes to accumulate) [172]. Beyond post-transcriptional regulation, sRNAs also function in transcriptional gene silencing through the RNA-directed DNA methylation (RdDM) pathway, which establishes methylation at stress-responsive promoters and transposable elements, preserving genome stability and adaptive plasticity [173,174,175].

Species-specific studies further illustrate the breadth of RNA-based regulation. In wheat, numerous non-coding RNAs, including lncRNAs (TalncRNA18, TalncRNA73, TalncRNA106, TalncRNA108), contribute to defense and abiotic stress responses [163,164,165,166,167]. In rice, diverse miRNAs and siRNAs regulate stress signaling, with 24-nt miRNAs and heterochromatic siRNAs (hc-siRNAs) guiding CHH methylation of transposable elements and stress-inducible promoters via RdDM during salt exposure [171,172,173,174,175,176,177]. Additionally, phasiRNAs have been linked to reproductive development and male sterility [176], while long siRNAs (lsiRNAs) target defense-related transcripts during pathogen attack [178]. Several functionally characterised lncRNAs in rice further modulate growth, development, and epigenetic reprogramming [179,180].

Emerging evidence highlights novel RNA regulators. Circular RNAs (circRNAs), for instance, act as “miRNA sponges,” sequestering active miRNAs and thereby indirectly enhancing the expression of stress-protective genes [181,182]. This additional regulatory layer emphasizes the expanding complexity of RNA-mediated control.

In summary, small RNAs and their interacting partners form a multilayered regulatory network that integrates transcriptional, post-transcriptional, and epigenetic processes. Through mechanisms such as RdDM, translational repression, and ncRNA-guided regulation, plants can rapidly adjust to salinity while also establishing stress memory and long-term adaptation. These insights highlight the potential of ncRNA-based approaches in breeding salt-tolerant crops.

### 3.4. Chromatin Remodelling as an Epigenetic Mechanism in Plant Stress Responses

The regulation of plant gene expression depends critically on chromatin remodelling. This process dynamically alters nucleosome positioning and density to control the accessibility of DNA regulatory elements such as promoters. This mechanism enables plants to fine-tune transcription in response to developmental cues and environmental stresses. While heat stress often induces global increases in chromatin accessibility, drought and salt stress generally maintain stable chromatin states, with remodeling occurring at specific loci of stress-responsive genes [183,184].

Two major groups of enzymes mediate chromatin remodelling:

- Chromatin remodelers, which use ATP-dependent, non-covalent mechanisms to reposition, evict, or restructure nucleosomes.
- Chromatin modifiers, which catalyze covalent changes to histones or DNA (e.g., acetylation, methylation, phosphorylation).

Plant remodelers are classified into four evolutionarily conserved families: SWI/SNF, ISWI, CHD, and INO80, all belonging to the SWI2/SNF2 superfamily of helicase-like ATPases.

- SWI/SNF family: Central to transcriptional reprogramming. In *Arabidopsis*, DDM1 promotes RdDM by enabling methyltransferase access to heterochromatin [185], while CLSY proteins guide siRNA biogenesis in tissue-specific RdDM targeting [186]. Other members, such as BRM and SYD, regulate hormonal signaling and stress responses, with BRM counteracting Polycomb repression and aiding stress-induced gene reactivation [187]. In rice, OsCHR4 has been linked to drought and salt tolerance by regulating stress-related transcription [188].
- ISWI family: Primarily maintains nucleosome spacing for transcriptional repression but can also facilitate RNA polymerase II access to promoters. In *Arabidopsis*, CHR11 and CHR17 sustain expression of heat-stress memory genes such as *HSA32* [189]. In maize, ZmCHB101 alters nucleosome density to influence both gene expression and alternative splicing during osmotic stress [150].
- CHD family: Functions are context-dependent, either repressing transcription (via association with HDACs or methyl-binding proteins) or promoting transcription by sliding nucleosomes to increase promoter accessibility [190,191].
- INO80 family: Associated with gene activation, DNA repair, and replication-coupled remodelling. These complexes safeguard genome stability, regulate developmental transitions, and contribute to stress-induced transcriptional reprogramming [192,193].

Chromatin remodelling also contributes to epigenetic stress memory. In *Arabidopsis*, BRM, CHR11/17, and the histone H3-binding factor FORGETTER1 (FGT1) maintain low nucleosome occupancy at stress-memory gene promoters, ensuring sustained transcription following heat exposure [189].

In salinity stress, remodeling intersects with hormone signaling. The phosphorylation of SnRK2 kinases and inhibition of PP2C phosphatases lead to inactivation of BRM, derepressing *ABI5* expression. Consistently, *brm-3* mutants display hypersensitivity to abscisic acid and reduced growth under saline conditions [194,195].

**Figure 4:**
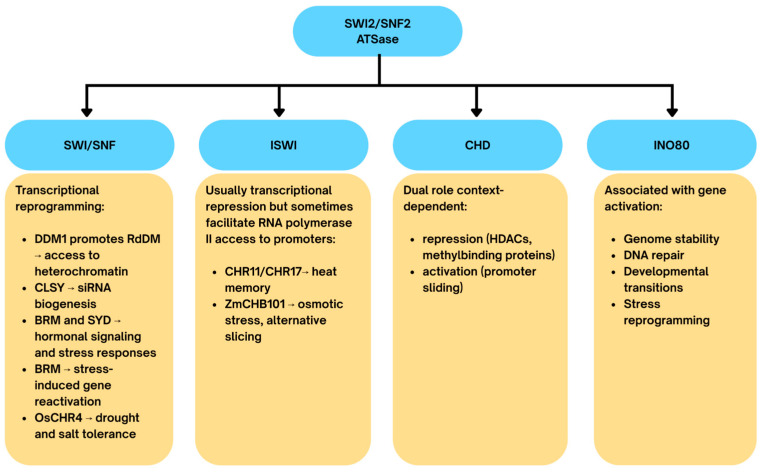
Families of ATP-dependent chromatin remodelers in plants. Chromatin remodeling in plants is mediated by ATP-dependent complexes belonging to the SWI2/SNF2 superfamily of helicase-like ATPases, which are classified into four evolutionarily conserved families: SWI/SNF, ISWI, CHD, and INO80. Members of the SWI/SNF family regulate transcriptional reprogramming, with DDM1 facilitating RdDM by enabling methyltransferase access to heterochromatin, CLSY proteins guiding siRNA biogenesis, and BRM counteracting Polycomb repression to activate stress-responsive genes; in rice, OsCHR4 is linked to drought and salinity tolerance. The ISWI family primarily controls nucleosome spacing, with *Arabidopsis* CHR11/CHR17 sustaining heat-stress memory and maize ZmCHB101 modulating nucleosome density during osmotic stress and alternative splicing. The CHD family exerts context-dependent roles, acting either as transcriptional repressors through interactions with HDACs and methyl-binding proteins or as activators by sliding nucleosomes to increase promoter accessibility. The INO80 family contributes to genome stability, DNA repair, developmental transitions, and stress-induced transcriptional reprogramming. Collectively, these remodeler families orchestrate chromatin dynamics to regulate plant development and adaptation under environmental stress conditions.

Additionally, chromatin remodeling rarely acts alone. Remodelers coordinate with histone acetyltransferases (HATs), histone deacetylases (HDACs), and DNA methylation machinery to ensure precise and stable regulation during stress responses [196,197]. Recent high-resolution methods such as ATAC-seq and nucleosome profiling are beginning to reveal stress-specific open chromatin landscapes in crops, including salt-responsive promoters in rice (Figure 3 and Figure 4) [198].

## 4. Strategies for Enhancing Salinity Tolerance in Rice: Progress and Ongoing Challenges

Salinity poses a major constraint to rice cultivation worldwide. While agronomic practices such as improved irrigation, soil amendments, and drainage systems have helped mitigate its impact, these approaches are often resource intensive and provide only partial or site-specific relief. Consequently, there has been a growing emphasis on integrating molecular and biotechnological tools with traditional breeding programs to accelerate the development of salt-tolerant rice. Advances in genomics, molecular markers, and genetic engineering now offer opportunities to dissect the complex physiological and genetic basis of salt tolerance, enabling the identification and targeted incorporation of beneficial traits into elite cultivars. This section outlines the major technological strategies, classical breeding, marker-assisted selection, and genetic engineering, and epigenetic regulation, that form the foundation of current efforts to enhance salt tolerance in rice.

### 4.1. Traditional Breeding Approaches for Salt Tolerance in Rice

#### 4.1.1. Conventional Breeding and Phenotypic Evaluation Under Salinity Stress

**Figure 5:**
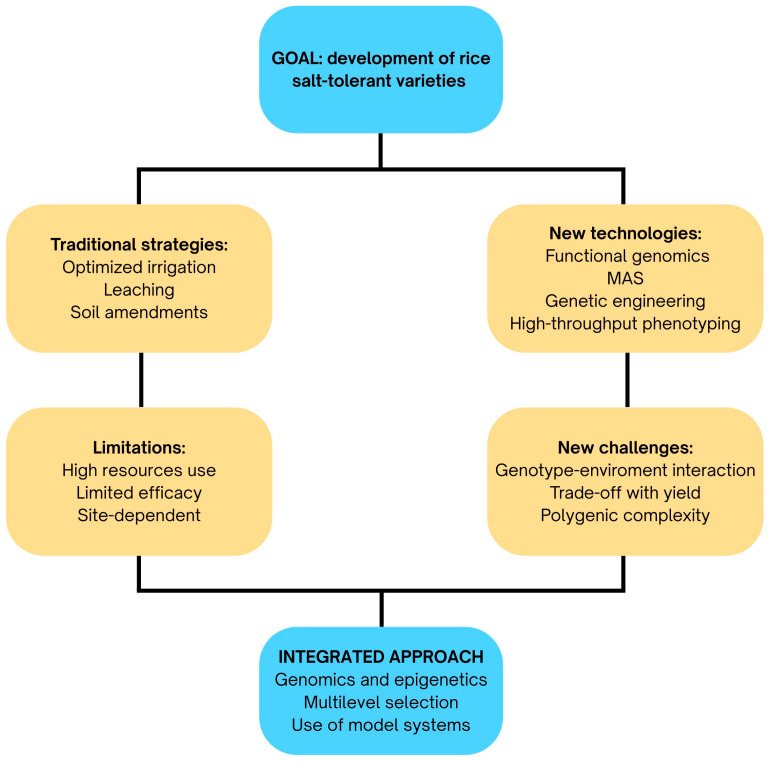
Conventional and biotechnological strategies for developing salt-tolerant rice varieties. This diagram summarises the approaches, challenges, and integrated solutions for improving rice salinity tolerance. (i) Traditional strategies: These include optimised irrigation, leaching, and soil amendments to mitigate salt accumulation. These methods are often resource-intensive, site-specific, and may have limited efficacy. (ii) New technologies: These encompass functional genomics, marker-assisted selection (MAS), genetic engineering, and high-throughput phenotyping to identify and incorporate stress-resilient traits. (iii) Limitations and challenges: Modern approaches face genotype–environment interactions, trade-offs with yield, and the polygenic nature of salt tolerance. (iv) Integrated approach: Combining genomics, epigenetics, multilevel selection, and model systems is increasingly recognized as essential to develop robust, salt-tolerant rice cultivars suitable for diverse agroecological contexts.

Conventional breeding has long served as the foundation for developing rice varieties better adapted to saline environments. The process typically involves two main steps: first, assembling or generating a diverse breeding population that exhibits variable responses to salt stress, and second, selecting individuals from segregating generations that combine strong salt tolerance with desirable agronomic traits [199]. The success of this approach largely depends on the availability of phenotypic variation for salt tolerance within accessible germplasm, which is often limited when working with elite but salt-sensitive varieties (Figure 5).

The evaluation of salt tolerance in breeding programs is commonly based on two parameters: the threshold level, or the highest salinity at which yield remains unaffected, and the slope, which reflects the percentage reduction in yield per unit increase in salinity beyond that threshold. For rice, the threshold is generally around 3.0 dS/m, with yields declining by approximately 12% for each additional dS/m [200]. However, plant responses to salinity are highly complex. They can vary with developmental stage, the type and concentration of salts, exposure duration, and environmental conditions such as soil pH, water availability, temperature, and light intensity [201]. Stress-induced epigenetic changes partly explain such variability. For example, salt stress can cause DNA methylation changes that alter gene expression even in closely related genotypes, as shown in rice [124]. Some of these epigenetic modifications persist across generations, creating a heritable “stress memory” that shapes adaptive responses, as demonstrated in *Arabidopsis thaliana* [13]. Because conventional selection may overlook such epigenetic variation, integrating epigenetic markers into breeding pipelines could improve the predictability of phenotypic outcomes [202].

#### 4.1.2. Traditional Breeding Strategies and Challenges in Developing Salt-Tolerant Rice

Traditional methods, including hybridization, varietal introduction, and induced mutation, have been employed to generate salt-tolerant lines, yielding some promising genotypes over time [203,204,205,206,207,208,209,210]. However, progress has been limited, largely due to the difficulty of identifying physiological traits that reliably predict tolerance, as these traits are typically controlled by multiple quantitative trait loci (QTLs) [211]. Additionally, many salt-tolerance traits are under epigenetic control rather than fixed genetic loci. Stress-responsive transcription factors and ion transporter genes are frequently regulated by histone modifications and small RNA pathways, leading to variable expression in different environments. This epigenetic variability partly explains why conventional selection can be inconsistent. Identifying and stabilizing beneficial “epialleles” could therefore complement traditional breeding approaches. Physiological traits such as ion regulation and osmotic adjustment have been proposed as screening criteria [212,213,214,215,216], and their strategic combination through selective breeding could enhance tolerance beyond current phenotypic limits [203,204]. Nonetheless, no varieties have been released based solely on these markers, and yield performance under controlled salinity conditions remains the most practical measure of tolerance [217]. Recent advances in high-throughput phenotyping and imaging platforms now offer scalable, non-destructive tools to quantify these traits more effectively.

#### 4.1.3. Growth Stage-Specific Sensitivity and Screening Strategies for Salinity Tolerance in Rice

Breeding for salinity tolerance in rice is further complicated by the crop’s variable sensitivity to salt stress across different growth stages. Rice tends to be more tolerant during germination, tillering, and late maturity, but it is highly susceptible during the early seedling and reproductive phases. Epigenetic regulation is highly developmental stage-specific. Evidence shows that methylation dynamics vary not only across tissues but also between vegetative and reproductive stages, shaping stage-specific stress responses [16]. In rice, for example, salt stress induces distinct methylation and gene expression profiles at the seedling versus reproductive stage, with significant consequences for growth and yield [218]. Moreover, flowering represents a particularly sensitive window: methylation and demethylation events triggered by salinity can disrupt reproductive development and directly impact fertility [219,220]. These findings highlight that profiling epigenetic marks at precise developmental phases could help breeders design more effective screening strategies, targeting tolerance mechanisms at the stages most critical for plant performance under saline conditions. Moreover, tolerance expressed at one stage often does not correlate with tolerance at another. For example, the rice line CN499-160-13-6 is sensitive during the seedling stage but demonstrates tolerance at flowering [221,222,223]. This lack of correlation necessitates a two-stage screening strategy: (i) initial screening at the seedling stage under controlled conditions to handle large populations, followed by (ii) evaluation of selected lines at the reproductive stage under field conditions [217]. These challenges underscore the importance of integrated screening pipelines that combine physiological, agronomic, molecular and epigenetic data across multiple growth stages.

#### 4.1.4. Genetic Diversity and Limitations of Salt Tolerance in Cultivated and Wild Rice Germplasm

A further limitation is that many salt-tolerant rice varieties identified to date exhibit poor yield or undesirable agronomic traits, making them unsuitable as donor lines for breeding programs. For example, while the cultivar Nona Bokra demonstrates strong tolerance, wild rice species tested so far—including relatives of both *O. sativa* and *O. glaberrima*—have not shown higher levels of tolerance than the best-performing cultivated types [224]. Moreover, the salt tolerance of many species within the *Oryza* genus remains largely unknown, representing untapped potential yet to be explored. Leveraging the wild *Oryza* gene pool through genomics and genome-wide association studies (GWAS) could help identify novel alleles that contribute to stress resilience.

Among the wild rice species, *Porteresia coarctata* (Takeoka), a halophyte native to salt marshes along the Bay of Bengal coast, exhibits remarkable salt tolerance, thriving in environments with up to 20% seawater salinity without adverse effects on growth [225]. In contrast, within the two major subspecies of cultivated rice, *indica* varieties generally display greater salt tolerance than *japonica*. This difference is often linked to more efficient sodium exclusion, enhanced potassium uptake, and maintenance of a favourable Na^+^/K^+^ ratio in shoot tissues [226]. Such differences highlight the substantial genetic variation in salt tolerance across rice cultivars [227,228,229,230]. Beyond DNA-sequence divergence, *japonica*, *indica*, and wild *Oryza* relatives exhibit distinct, background-specific DNA methylation landscapes, including at promoters, that associate with regulatory and expression differences [231,232]. Under salinity, promoter-proximal epigenetic states can directly gate the inducible expression of ion-transporter genes—for example, a DNA-methylation reader–chaperone–transcription factor complex activates the Na^+^ transporter OsHKT1;5 in rice, illustrating how promoter context enables stronger stress-induced transcription [233]. Together, these findings support the conservation and exploitation of epigenetic diversity within germplasm, alongside genetic diversity, to enhance stress tolerance without compromising agronomic performance [234].

#### 4.1.5. Leveraging Salt-Tolerant Landraces and the Saltol QTL for Rice Improvement: Progress, Limitations, and Future Directions

Despite numerous attempts to exploit traditionally recognised tolerant lines such as Nona Bokra, Pokkali, SR26B, and Kalarata, these varieties still do not match the high level of tolerance observed in P. coarctata [200]. A major drawback of these landraces lies in their poor agronomic traits, including tall plant stature, low grain yield, photosensitivity, and substandard grain quality [200,201]. Among them, Pokkali is particularly noteworthy for its comparatively higher salt tolerance, making it a valuable donor in breeding programs [235]. Notably, a major quantitative trait locus (QTL), named Saltol, was identified in Pokkali on chromosome 1. This QTL is associated with tolerance during the vegetative growth stage and accounts for up to 80% of the phenotypic variation under salt stress [92,222,236,237]. Saltol has since been introgressed into several elite rice varieties through marker-assisted selection (MAS), highlighting the practical relevance of QTL-based breeding.

An improved line, FL478 (derived from a cross between Pokkali and IR29), exhibits better agronomic performance, including shorter stature, earlier flowering, and photoperiod insensitivity, while retaining strong seedling-stage salt tolerance. FL478 also maintains a lower Na^+^/K^+^ ratio than either of its parents, and its vigorous tillering and superior potassium retention under salinity stress make it a promising resource for developing salt-tolerant rice varieties, particularly during early growth stages [238,239]. Nevertheless, additional QTLs beyond Saltol are required to enhance tolerance at the reproductive stage, which remains a critical bottleneck in breeding for salinity resistance across the entire life cycle. The limited effectiveness of Saltol in the field may be linked to its epigenetic regulation. DNA methylation and histone modifications under salt stress are highly dynamic and organ-specific, strongly affecting gene expression at the seedling stage [47,124]. In contrast, reproductive development relies on distinct QTLs and epigenetic controls that can suppress stress-responsive genes, reducing tolerance during flowering [240,241]. Epigenomic mapping of Saltol and related loci could therefore guide QTL pyramiding by clarifying stage-specific regulation.

### 4.2. Marker-Assisted Selection and QTL-Based Breeding for Salinity Tolerance in Rice: Advances, Applications, and Challenges

Advances in molecular genetics have enabled substantial progress in improving rice salinity tolerance through marker-assisted selection (MAS; Figure 5). A major breakthrough was the identification of the Saltol QTL on chromosome 1 from a cross between IR29 and Pokkali, which explains over 70% of the variation in sodium uptake and has since been introgressed into elite but salt-sensitive cultivars [221]. Because salt tolerance is a polygenic trait, pinpointing key QTLs such as Saltol is essential for dissecting the genetic basis of stress responses and facilitating their deployment in breeding programs.

A range of approaches have been used for QTL discovery, including classical map-based cloning [242], microarray-based gene expression profiling [243], and integrative strategies that combine genetic mapping with transcriptomics [244,245]. These efforts have identified numerous QTLs associated with salt tolerance. For example, a study using an F_2_ population derived from the tolerant line CSR27 and the susceptible MI48 identified 25 significant QTLs across chromosomes 1, 2, 3, and 8. These loci were linked to traits such as seedling injury scores, ion (Na^+^, K^+^, Cl^−^) concentrations in leaves and stems, and reproductive-stage stress tolerance, based on 79 SSR and EST markers spanning the rice genome [246].

Integration of mapping with bulked transcriptome profiling has further refined QTL discovery, associating intervals on chromosomes 1, 8, and 12 with ion regulation, and identifying a QTL for spikelet fertility under salinity stress on chromosome 8. However, the detection of more than 2600 genes within these regions underscores the challenge of pinpointing functional candidates [245]. Parallel studies in recombinant inbred lines (RILs) from the IR29 × Pokkali cross have confirmed Saltol and revealed additional QTLs for salinity tolerance. In contrast, the development of backcross and near-isogenic lines has enhanced validation and facilitated practical breeding applications [247].

Functional characterisation of Saltol has highlighted its central role in maintaining Na^+^/K^+^ balance in shoots, a key mechanism of rice salt tolerance [248,249]. These mechanistic insights have increased the precision of MAS, which offers clear advantages over conventional breeding: it can be applied at any growth stage, is minimally influenced by environmental variation, avoids dominance effects, and is efficient even in early generations.

Building on MAS, marker-assisted backcrossing (MABC) has been successfully used to introgress Saltol into elite cultivars. Improved versions of widely grown varieties such as PB1121 and PB6 [250], AS996 [251], Bac Thom 7 [252,253], and BRRI Dhan-49 [254] now combine high yield potential with enhanced resilience to salinity stress. These cases illustrate how MABC accelerates the integration of major QTLs into breeding pipelines.

Despite these advances, MAS and MABC face important limitations. A major challenge is linkage drag, where large chromosomal segments bring undesirable traits alongside the target QTL [255]. Furthermore, the expression and effectiveness of QTLs are influenced by both genetic background and environmental conditions, limiting their stability across diverse production environments [256]. Epigenetic variability, particularly via DNA methylation, may underlie QTL instability across environments by modulating trait expression in a context-dependent manner. For instance, in cotton, over 5 million cis-meQTLs have been identified, many of which associate with fiber yield and quality traits independently of genetic variants, revealing the value of methylation data for breeding beyond conventional GWAS approaches [257]. Incorporating epigenetic markers into MAS pipelines could thus enhance predictive accuracy for QTL stability. Moreover, epigenetic variation appears to evolve more rapidly than genetic variation, potentially representing a resource for selection that could enhance adaptability and trait improvement [258]. (Figure 5).

### 4.3. Genetic Engineering for Salinity Tolerance in Rice: Transgenic Approaches, Challenges of Gene Silencing, and Future Prospects

Genetic engineering has emerged as a powerful complement to conventional and molecular breeding, allowing the precise introduction or modification of genes associated with stress tolerance without altering desirable agronomic traits [101]. The development of efficient *Agrobacterium tumefaciens*-mediated transformation systems for rice has significantly expanded the scope for targeted improvement [259].

Transgenic approaches to enhance salinity tolerance have focused on several key functional categories: (i) osmoprotectants such as proline and glycine betaine, (ii) antioxidants that mitigate reactive oxygen species (ROS), (iii) ion transporters that regulate Na^+^ exclusion and K^+^ uptake, and (iv) regulators of programmed cell death and transcriptional networks (Table 2). By integrating these mechanisms, genetic engineering aims to replicate the multilayered tolerance strategies found in naturally tolerant genotypes such as Pokkali, which employs vacuolar Na^+^ sequestration alongside enhanced antioxidant activity [95,260]. However, no transgenic rice cultivar with improved salt tolerance has yet been released for commercial cultivation, underscoring the persistent challenges in translating laboratory success into field performance.

Two major barriers limit the success of transgenic interventions. First, the genetic architecture of salt tolerance is highly complex, with multiple pathways operating across developmental stages. The weak correlation between tolerance at the seedling and reproductive phases highlights why single-gene interventions are often insufficient. Consequently, pyramiding multiple loci, rather than relying on a single transgene, is increasingly viewed as a more effective strategy to ensure tolerance throughout the rice lifecycle.

Second, gene silencing poses a significant obstacle to transgene stability. Both transcriptional gene silencing (TGS) and post-transcriptional gene silencing (PTGS) can reduce or abolish transgene expression, with variable effects observed across different lines and generations [261,262,263,264,265,266]. While TGS, typically driven by DNA methylation of promoter regions, is largely irreversible, PTGS—mediated by small RNAs targeting transcripts—is more dynamic but equally disruptive. These silencing mechanisms, frequently reported in rice [267,268,269], complicate reliable trait expression. A key obstacle to stable transgene expression is epigenetic silencing, in which small-RNA–guided pathways (RdDM) deposit repressive chromatin and DNA methylation at the introduced locus, ultimately shutting down expression [270,271,272]. This underscores that engineering strategies must account for local chromatin state and epigenetic feedback. As an alternative to adding new genes, CRISPR-based epigenome editing can reprogram endogenous loci: dCas9–TET1 enables targeted demethylation and promoter activation, while dCas9–SunTag fusions can deposit methylation to tune expression with locus specificity [273,274].

To counteract these effects, researchers have explored strategies such as extrachromosomal expression systems, the inclusion of introns, artificial chromosomes, and viral suppressors of silencing. More recently, CRISPR/Cas-based editing offers precise, integration-free modifications that may help reduce silencing and improve expression stability. Yet despite the promise of genetic engineering, widespread adoption remains limited by regulatory barriers and public concerns, particularly the risks of off-target effects and uncertainties surrounding long-term consequences

In parallel, genome-wide association studies (GWAS) have provided a complementary strategy for dissecting the genetic basis of salinity tolerance. By leveraging natural variation across diverse germplasm, GWAS enables the identification of loci with finer resolution than traditional linkage mapping. These studies have identified multiple genomic regions associated with ion homeostasis, ROS detoxification, and developmental plasticity. However, GWAS is constrained by genotype × environment interactions, population structure biases, and the limited durability of single-locus associations. As such, its most promising application lies in combination with genomic selection and functional validation, ensuring that identified loci contribute robustly to breeding and biotechnological efforts.

Together, genetic engineering and genome-wide approaches represent cutting-edge tools for improving salinity tolerance in rice. Yet, both face substantial challenges that underscore the need for integrative strategies—combining molecular, genomic, and epigenetic interventions with precision breeding to achieve stable, field-ready salt-tolerant cultivars. Epigenetic approaches provide flexibility for crop improvement, but marks can be environmentally unstable and show variable heritability, as seen in stress-induced methylation changes in rice [275]. Moreover, cost-effective, high-throughput epigenomic profiling remains limited, despite progress with improved ChIP-based methods. Future breeding strategies should integrate epigenomic data with genomic selection and phenotyping pipelines to deliver saline-tolerant cultivars [276].

**Table 2:** Representative genes introduced into rice via genetic engineering to enhance salinity tolerance, organized by functional category. This table lists genes grouped by their primary physiological role, the mechanism through which they confer salt tolerance, and key references.

Functional Category	Gene Symbol(s)	Function/Mechanism	Reference(s)
**Osmoprotectants**	*P5CS*, *BADH*, *TPS1*	Proline, glycine betaine, trehalose synthesis	[277,278,279]
**Antioxidants**	*SOD*, *CAT*, *GST, GS2*	Detoxification of ROS, reduction in oxidative damage	[280,281,282,283]
**Ion Transporters**	*HKT1;5*, *NHX1*, *SOS1*	Na^+^ exclusion, vacuolar sequestration, Na^+^/H^+^ antiport	[284]
**LEA/Chaperones**	*LEA3-1*, *HSP70*	Protection of proteins and membranes under stress	[285,286,287,288]
**Transcription Factors**	*DREB1A*, *NAC*	Activation of stress-responsive genes	[289,290]
**PCD Regulators**	*AtBAG4*, *p35*	Suppression of programmed cell death under salt stress	[285,288]
**Signal Transduction Genes**	*CDPK*, *MAPK*	Calcium and ABA signaling pathways	[291,292]

## 5. Harnessing Wild Oryza Genetic Resources for Salinity Tolerance and Sustainable Rice Improvement

Despite significant challenges in improving salinity tolerance in rice, such as the limited availability of suitable parental lines for conventional breeding and the complex, polygenic nature of the trait that constrains the effectiveness of marker-assisted selection and genetic engineering, emerging strategies offer new opportunities. Wild rice species, for example, serve as desalinization crops and reservoirs of valuable genetic variation that can be harnessed for breeding and biotechnological interventions. Additionally, genome editing tools provide a precise means to enhance salt tolerance in elite cultivars.

Soil salinisation is an increasing global concern, affecting even regularly irrigated lands. Each year, roughly two million hectares of irrigated fields become unsuitable for cultivation due to excessive salt accumulation [293]. A common remediation method is leaching, where large volumes of water are used to flush salts from the root zone [294]. Rice paddies, with their standing water, naturally facilitate this process, and *Oryza sativa*, thanks to its adaptation to flooded conditions, is often recommended as a desalinization crop for reclaiming moderately salt-affected soils [295].

Wild rice relatives offer a critical reservoir of genetic diversity for developing salt-tolerant and nutrient-rich rice cultivars, addressing both soil salinization and climate-related challenges. The International Rice Research Institute (IRRI) recognizes 25 wild *Oryza* species in addition to the two cultivated species, *O. sativa* and *O. glaberrima* [296]. Among these, A-genome species, such as African *O. longistaminata* and *O. barthii*, South American *O. glumaepatula*, and Austral-Asian *O. rufipogon*, *O. nivara*, and *O. meridionalis*, are particularly valuable due to their genetic compatibility with cultivated rice.

Several wild species harbour alleles associated with salinity tolerance. For example, *O. rufipogon*, the progenitor of cultivated *O. sativa*, contains quantitative trait loci (QTLs) that enhance salt tolerance when introgressed into elite cultivars [297]. Coastal species like *O. coarctata* provide models for studying natural adaptations to high salinity [298]. Beyond abiotic stress, wild species also offer improved nutritional profiles. Australian wild rice populations, including *O. meridionalis* and *O. rufipogon*, have grains with ~10% protein and elevated mineral content, compared to ~7% in typical *O. sativa* varieties [299,300,301].

Genomic advances further highlight the potential of wild *Oryza*. Whole-genome sequences are available for multiple species, revealing extensive variation in genes associated with stress responses, reproduction, and development [302,303,304]. Northern Australian species, such as *O. meridionalis*, show particularly high divergence, reflecting long-term geographic isolation and adaptation to heterogeneous floodplain environments [301,305,306,307,308,309]. Studies also suggest that some Australian wild rice may carry adaptive alleles for nutrient-poor soils and other local stressors [310].

Recent research highlights that epigenetic regulation plays a pivotal role in the adaptation of *Oryza* species to environmental stresses such as salinity. DNA methylation and histone modifications have been shown to regulate the expression of ion transporters, contributing to osmotic balance and enhanced salt tolerance in rice and related species [36,311]. These reversible modifications provide plants with a dynamic mechanism to fine-tune stress-responsive genes without altering the DNA sequence itself. In parallel, small RNAs have emerged as crucial regulators of stress responses in wild rice relatives, such as *O. rufipogon*, where several microRNAs are differentially expressed under saline conditions and likely contribute to adaptive plasticity [312]. Together, these epigenetic mechanisms represent a valuable reservoir of variation, with potential application in breeding programs aimed at improving stress tolerance by exploiting stable epigenetic variants.However, the potential of wild rice is threatened by habitat loss and genetic contamination from expanding cultivation. Gene flow from domesticated rice has already compromised populations in China and elsewhere [313,314,315,316]. In northern Australia, rice cultivation is expanding near pristine wild habitats, underscoring the urgency of conservation measures, such as buffer zones and pollen traps, to safeguard these genetic resources [317,318]. Conservation is not only about protecting nucleotide diversity but also about preserving epigenetic variation moulded by local environments. Reviews in ecological epigenetics show that DNA methylation and other epigenetic marks can shape ecologically relevant phenotypes and contribute to adaptive “stress memory,” making epigenomic information directly relevant to conservation practice [319,320].

Because epigenetic patterns, particularly stress-adaptive methylation states, may be population-specific and environmentally conditioned, the erosion of wild habitats risks losing adaptive epialleles that cannot be inferred from DNA sequence alone. Consequently, ex situ and in situ strategies should pair genomic with epigenomic characterisation to capture both allelic and epiallelic components of adaptive potential [320,321].

In rice, wild *Oryza* populations are a reservoir of adaptive traits, including loci that enhance salinity tolerance; empirical work demonstrates that genes from wild rice can markedly improve salt tolerance in cultivated backgrounds [322]. Strategic “mining” of wild *Oryza* should therefore combine genomic scans for adaptive loci with epigenomic profiling to detect stress-responsive epialleles contributing to salinity tolerance [321]. At the same time, maintaining wild-rice biodiversity remains critical to sustain this reservoir for future breeding and food security [323].

## 6. From Plant Transformation to Genome Editing: Advances in Precision Engineering of Rice for Salinity Tolerance

Advances in plant transformation technologies since the 1980s have enabled the development of genetically modified crops, including efforts to enhance salinity tolerance in rice [324]. Over time, plant transformation methods have advanced to allow for more precise control over transgene integration, targeted DNA sequence modifications, and transformation systems that can minimise the introduction of selection markers into the genome. Building on these advances, genome editing technologies now enable precise, site-specific changes in plant genomes without the stable integration of foreign DNA. These approaches induce double-stranded breaks (DSBs) at defined genomic sites, which are then repaired by the plant’s endogenous DNA repair pathways—most commonly non-homologous end joining (NHEJ) and, less frequently, homologous recombination (HR). A major milestone in this field was the development of sequence-specific nucleases (SSNs), including zinc-finger nucleases, TALENs, and CRISPR/Cas systems, which allow efficient and accurate induction of DSBs at predetermined genomic locations. Beyond introducing sequence variation, CRISPR platforms can be repurposed for epigenome editing by tethering dCas9 to methylation writers or erasers, thereby reprogramming DNA methylation at specific loci without altering the underlying sequence. For example, dCas9–SunTag–DNMT3A induces robust, kilobase-scale CpG methylation with minimal global off-target effects and represses transcription [325]. In plants, locus-specific demethylation reactivates genes: TET1cd fusions produced heritable late-flowering when targeted to the *FWA* promoter in *Arabidopsis* (ZF–TET1cd), and the same study adapted CRISPR/dCas9 SunTag–TET1cd for precise demethylation with limited off-target changes [273]. Such programmable epigenetic control is pertinent to salinity tolerance, where tolerant vs. sensitive rice cultivars show divergent and flexible DNA methylation responses—including promoter and gene-body changes—correlated with stress-responsive gene expression under salt stress [124,137].

### 6.1. CRISPR/Cas Genome Editing in Rice: Simplicity, Precision, and Challenges in Developing Transgene-Free Crops

Several systems are available to introduce precise double-stranded breaks (DSBs) for genome editing, including meganucleases, zinc finger nucleases (ZFNs), and transcription activator-like effector nucleases (TALENs). These tools rely on engineered proteins that recognize specific DNA sequences. While effective, they are relatively complex and require extensive customization for each new target site (Figure 4).

The development of the CRISPR/Cas system has revolutionised genome editing due to its simplicity, versatility, and efficiency. Derived from bacterial defence mechanisms that target foreign DNA, CRISPR/Cas uses a short RNA molecule (CRISPR RNA or crRNA) to guide the Cas9 nuclease to a specific genomic site, where it introduces a DSB [326]. Unlike ZFNs or TALENs, retargeting CRISPR/Cas requires only modification of a ~20-base-pair guide RNA, eliminating the need for labour-intensive protein engineering.

CRISPR/Cas offers several key advantages over earlier tools: (i) broader range of potential target sites, (ii) higher delivery efficiency, since guide RNAs are much smaller than protein-based nucleases, and (iii) high specificity and the ability to edit multiple genes simultaneously (multiplexing). These advantages have made CRISPR/Cas the preferred method for editing plant genomes and improving crops. For example, editing the rice OsERF922 gene via CRISPR/Cas9 enhanced resistance to rice blast without integrating foreign DNA. In these studies, edited lines identified in the T1 generation displayed markedly reduced lesion development, and the edits were heritable and transgene-free as early as the first generation [327,328]. A central goal in genome editing is to achieve precise, site-specific modifications without introducing extraneous DNA. A central goal of genome editing is to achieve precise, site-specific modifications without introducing extraneous DNA. Unlike traditional transgenic methods that insert foreign genetic material, genome editing uses endogenous repair pathways like homology-directed repair (HDR) to modify native sequences. A persistent challenge, however, is the inadvertent incorporation of foreign DNA during transformation, which subsequently must be removed through breeding and selection. Overcoming this bottleneck is critical to fully realising the potential of genome editing for crop improvement and broader acceptance of genome-edited plants. Beyond nuclease-mediated editing, CRISPR can be re-engineered for transcriptional and epigenetic modulation without introducing double-strand breaks. In plants, dCas9-based epigenome editing has been used to erase DNA methylation at specific loci and activate gene expression, including via a CRISPR/dCas9 SunTag–TET1 system in *Arabidopsis* [273].

In parallel, CRISPRa fusions to chromatin writers can fine-tune stress-responsive genes; for instance, a dCas9–HAT activator elevated endogenous *AREB1* and improved drought tolerance without altering the DNA sequence [329].

Importantly for salinity, DNA methylation regulates the rice Na^+^ transporter gene *OsHKT1;5* through a methylated upstream MITE bound by the methylation reader OsSUVH7 within a BAG4–MYB–SUVH complex that activates *OsHKT1;5* under salt stress [233].

Taken together, these findings indicate that dCas9 fusions with DNA demethylases or histone acetyltransferases could be deployed to activate salinity-tolerance genes in rice by altering local chromatin or methylation states—an approach that is programmable and potentially reversible; its feasibility in rice is supported by successful CRISPR-guided targeted demethylation producing heritable epialleles [273,329,330].

### 6.2. Toward Transgene-Free Genome Editing in Rice: Emerging Tools and Approaches

Several strategies have been developed to minimise or eliminate unintended DNA integration during genome editing. One approach involves using *Agrobacterium tumefaciens* strains with defective VirD2 proteins, which are key components of the T-DNA transfer machinery. These modified strains drastically reduce stable DNA integration while still supporting transient gene expression, making them a promising tool for DNA-free genome editing in rice [286].

Alternative strategies focus on biolistic (gene gun) methods. Chemically modifying the DNA that coats gold particles can limit its nuclear entry, allowing temporary expression without stable incorporation. Similarly, delivering single-stranded DNA, which is less prone to genomic integration, offers another route for creating non-integrative edits (Table 3). Although these approaches are still being optimised, they demonstrate feasible paths toward efficient, transgene-free genome editing. This capability is especially valuable as regulatory frameworks for genome-edited crops continue to evolve, potentially distinguishing them from conventional genetically modified organisms. Beyond regulatory advantages, the ability to make precise edits without foreign DNA integration enhances the development of improved rice cultivars. It provides a powerful tool for fundamental research on gene function and plant development. Importantly, DNA-free genome editing platforms are well-suited to epigenome editing in rice because protein-only CRISPR ribonucleoproteins can be delivered directly into rice zygotes to generate edits without transgene integration [331]. In plants, dCas9-based epigenetic effectors can precisely write or erase DNA methylation at defined loci, for example, SunTag-guided DRM methyltransferase modules that install locus-specific methylation with meiotic heritability, and dCas9-TET1 systems that induce targeted demethylation and gene reactivation, establishing both programmability and the possibility of reversibility [273,274]. In rice specifically, targeted DNA demethylation has produced heritable epialleles, showing that locus-specific epigenetic states can be engineered and transmitted [330]. Because salt tolerance in rice is linked to DNA methylation dynamics at stress-responsive pathways (e.g., jasmonate-related genes), DNA-free, dCas9-mediated “epigenetic engineering” of these loci offers a promising route to stabilise salinity tolerance while retaining the option of reversion [273,332].

## 7. Conclusions

The complexity of salinity tolerance in rice, being a polygenic trait strongly influenced by environmental variability, requires integrated solutions that combine genetic, epigenomic, and agronomic approaches. Recent advances in genome editing enable precise, targeted modifications of endogenous genes without introducing foreign DNA, reducing regulatory hurdles and minimizing transgene silencing. These technologies, alongside insights from wild rice species, offer complementary avenues: wild relatives harbour naturally occurring alleles for salt tolerance and other favourable agronomic traits, serving both as gene donors and potential direct cultivars in saline or flood-prone environments.

Epigenomic tools provide an additional layer of control, enabling the modulation of gene expression without altering the DNA sequence. By mapping stress-responsive epigenetic marks and integrating this information with systems biology models of rice stress-response networks, researchers can identify regulatory hubs and predict effective combinations of tolerance genes. Such holistic approaches enhance the precision and efficiency of breeding and genome-editing programs, increasing the likelihood of field-ready outcomes.

Beyond genetic interventions, low-cost agronomic strategies, such as harnessing salt-tolerant rhizobacteria, optimising irrigation, and applying soil amendments, can synergise with genetic improvements, particularly in resource-limited systems. These practices offer scalable, context-sensitive solutions while supporting soil reclamation, as rice paddies under flooded conditions naturally leach salts from degraded soils.

To translate laboratory and greenhouse advances into impactful field solutions, future strategies should integrate multiple innovations: pyramiding complementary tolerance genes, leveraging pan-genomics and speed breeding to exploit natural variation, and employing artificial intelligence for predictive breeding. Attention must also be given to regional constraints, adoption challenges, and yield fitness trade-offs, ensuring that improvements are both resilient and practical.

Call to Action: Policymakers, funding agencies, and research consortia should prioritize integrated programs that combine genome editing, epigenomic insights, wild rice genetic diversity, and sustainable agronomic practices. This multi-pronged approach will accelerate the development of rice cultivars that can thrive under salinity stress, thereby supporting global food security and environmental sustainability.

## Figures and Tables

**Table 1:** Key genes, transporters, and regulatory proteins involved in ion homeostasis and Na^+^ compartmentation in rice under salt stress. Arrows in the table indicate the direction of effect: ↑ = increase or enhancement of the indicated parameter (e.g., K^+^ uptake, salt tolerance); ↓ = decrease or reduction in the stated parameter (e.g., cytosolic Na^+^, gene expression). Gene/Protein: Name of the gene or protein involved in ion transport or signaling. Function: Primary molecular role, including type of transporter or regulatory protein. Localisation: Cellular or tissue compartment where the protein predominantly functions. Phenotypic Effect: Observable physiological or biochemical outcome in the plant under salt stress. Key Notes: Additional context, regulatory interactions, or cultivar-specific observations relevant to salt tolerance.

Gene/Protein	Function	Localization	Phenotypic Effect	Key Notes
OsSOS1	Na^+^/H^+^ antiporter (Na^+^ efflux)	Plasma membrane	↓ Cytosolic Na^+^, ↑ salt tolerance	Activated by OsCBL4–OsCIPK24 (SOS pathway); truncated C-terminal variants show higher activity [87]
OsNHX1–4	Na^+^/H^+^ antiporters (vacuolar sequestration)	Tonoplast (vacuolar membrane)	Vacuolar Na^+^ compartmentation, cytoplasmic protection	Overexpression enhances salt tolerance in rice and maize [85]
OsHKT1;5	Na^+^ transporter (xylem retrieval)	Vascular tissues	↓ Na^+^ in shoots, protection of younger leaves	Higher expression in tolerant cultivars [92]
OsHAK10/OsHAK16	High-affinity K^+^ transporters	Roots and leaves	↑ K^+^ uptake, improved K^+^/Na^+^ ratio	Key for ionic rebalancing under salinity [84]
OsAKT1	K^+^ inward-rectifying channel	Root plasma membrane	↓ expression in sensitive lines; improved tolerance when maintained	Downregulated by salt stress; induced by OrbHLH001 overexpression [89]
OSA3	Vacuolar H^+^-ATPase	Tonoplast	Generates H^+^ gradient to power NHX transport	Strongly induced in salt-tolerant mutants [86]
OsCLC1	Cl^−^ channel/H^+^/Cl^−^ antiporter	Vacuole, Golgi, chloroplast	Cl^−^ detoxification, charge balance	Upregulated in *Pokkali*, repressed in *IR29* [91]
OsCBL4 (SOS3)	Ca^2+^ sensor	Cytosol	Activates OsCIPK24 → OsSOS1	Part of SOS signaling; senses salt-induced Ca^2+^ spike [86]
OsCIPK24 (SOS2)	Protein kinase	Cytosol	Phosphorylates and activates OsSOS1	Ca^2+^-dependent via OsCBL4; key for Na^+^ efflux [86]

**Table 3:** Strategies for DNA-free or low-integration genome editing in rice. This table summarises key approaches, their mechanisms, the associated risk of foreign DNA integration, and the main advantages.

Approach	Description	Integration Risk	Advantages
***Agrobacterium* VirD2-defective strains** [333]	Use of *A. tumefaciens* strains with mutations in VirD2 protein to impair T-DNA integration	Very low	Supports transient expression without integration
**Chemically modified DNA in biolistics** [333]	Coating gold particles with DNA modified to reduce nuclear entry and prevent integration	Low	Temporary expression, avoids genomic insertion
**Single-stranded DNA (ssDNA) delivery** [334,335]	Delivery of ssDNA instead of dsDNA via biolistics to reduce integration likelihood	Very low	Lower chance of stable integration
**Ribonucleoprotein (RNP) complexes** [336]	Direct delivery of CRISPR/Cas9 protein and guide RNA as a complex without any DNA	None	Truly DNA-free, no insertion risk
**Protoplast electroporation** [337,338]	Introduction of genome editing components into protoplasts using electric pulses	Variable	Efficient delivery, useful in controlled settings

## Data Availability

In this work, no new data were created.

## Abbreviations

CHH: CHH methylation context; CRISPR, clustered regularly interspaced short palindromic repeats; DNMTs, DNA methyltransferases; DSB, double-strand break; HATs, histone acetyltransferases; HDACs, histone deacetylases; HKT, high-affinity potassium transporter; MAS, marker-assisted selection; PCD, programmed cell death; QTL, quantitative trait locus; RdDM, RNA-directed DNA methylation; ROS, reactive oxygen species; SOD, superoxide dismutase; TALENs, transcription activator-like effector nucleases; TGS, transcriptional gene silencing; ZFNs, zinc finger nucleases.
